# Physical Activity Is Associated With Improved Eating Habits During the COVID-19 Pandemic

**DOI:** 10.3389/fpsyg.2021.664568

**Published:** 2021-04-12

**Authors:** Diego G. D. Christofaro, André O. Werneck, William R. Tebar, Mara C. Lofrano-Prado, Joao Paulo Botero, Gabriel G. Cucato, Neal Malik, Marilia A. Correia, Raphael M. Ritti-Dias, Wagner L. Prado

**Affiliations:** ^1^School of Technology and Sciences, São Paulo State University (Unesp), Presidente Prudente, Brazil; ^2^Department of Nutrition, Center for Epidemiological Research in Nutrition and Health, School of Public Health, Universidade de São Paulo (USP), São Paulo, Brazil; ^3^Independent Researcher, San Bernardino, CA, United States; ^4^Human Movement Science and Rehabilitation Graduation Program, São Paulo Federal University, Santos, Brazil; ^5^Department of Sport, Exercise and Rehabilitation, Northumbria University, Newcastle upon Tyne, United Kingdom; ^6^California State University, San Bernardino, CA, United States; ^7^Post-graduate program in Medicine, Universidade Nove de Julho, São Paulo, Brazil; ^8^Post-graduate Program in Rehabilitation Science, Universidade Nove de Julho, São Paulo, Brazil

**Keywords:** COVID-19, dietary pattern, exercise, food intake, lyfestyle, lockdown, developing countries, health

## Abstract

The aim of this study was to analyze the association between physical activity and eating habits during the COVID-19 pandemic among Brazilian adults. A sample of 1,929 participants answered an online survey, however 1,874 were included in the analysis. The impact of the COVID-19 pandemic on eating habits was assessed inquiring about participants' intake of fruits, vegetables, fried foods, and sweets during the pandemic. Physical activity was assessed by asking participants about their weekly frequency, intensity and number of minutes/hours engaging in structured physical activities per week. Participants were then stratified into categories based on moderate-to-vigorous intensity (0–30; 31–90; 91–150; 151–300; and >300 min/week) and into active (≥150 min) or inactive (<150 min). Increased sweets consumption was the most commonly reported change to eating habits (42.5%), followed by an increase in the consumption of vegetables (26.6%), fruits (25.9%), and fried foods (17.9%). Physical activity practice was related to lower consumption of fried foods (OR = 0.60; *p* < 0.001) and sweets (OR = 0.53; *p* < 0.001). A cluster analysis revealed subjects with higher the level of physical activity was more likely to follow a healthy diet (*p* < 0.001). Thus, physical activity was positively associated with healthier eating habits. Health authorities must recommend regular physical as a strategy to improve overall health during the COVID-19 pandemic. Future studies should address the physical activity interventions to improve health status during a pandemic.

## Introduction

The world has undergone a major transformation due to the epidemic caused by COVID-19 (Wang, 2020), which is a respiratory syndrome caused by the SARS-CoV-2 virus. COVID-19 is highly contagious. As of May 2020, more than 400,000 Brazilians have been diagnosed with COVID-19. To counteract the spread of the virus, several states within the country have begun mandating social distancing and self-quarantine (World Health Organization., 2020).

In May 2020, the Brazilian government recommended social distancing. Many gymnasiums were closed during this period. In addition, telecommuting was encouraged and schools were closed. Such actions may have contributed to changes in physical activity levels and dietary habits of participants (Rogers et al., 2020).

Prior to the COVID-19 pandemic, some studies showed that regular physical activity were associated with healthier eating habits (Pavičić ŽeŽelj et al., 2019). Further, physical activity and healthful eating habits were also associated with improvements in immune system function (Defagó et al., 2019; Weyh et al., 2020), which in turn, may reduce one's risk for developing COVID-19 symptoms or complications (Hamer et al., 2020).

However, Italian researchers conducted a study simultaneously with the present study and discovered that mandated restrictions during the COVID-19 pandemic, such as social distancing, contributed to the reduction of regular physical activity (Franco et al., 2021). In addition, Cheikh Ismail et al. (2020) analyzed data from countries in the Middle East and north Africa during the COVID-19 pandemic discovered an increase in the number of meals consumed and that ~50% of participants did not consume fruits on a daily basis. However, the relationship between physical activity and eating habits during the pandemic is unknown.

Our aim was to analyze the association between physical activity and eating habits during the COVID-19 quarantine among Brazilian adults.

## Methods

### Sample Study Design

The study was conducted in Brazil between May 5th and May 17th, 2020. At the time of data collection, public health measures were in place. Among these measures was the incentive for professionals to work from home when possible and the suspension of in-person classes among many schools which transitioned to online learning. Participants were invited through social media to answer an online questionnaire using the Google Forms platform (Mountain View, CA, USA). The inclusion criteria were adults over the age of 18 years that answered all of the survey questions. This study was approved by the Universidade Nove de Julho' Ethics Committee prior to data collection (CAAE #30890220.4.0000.5511). Participants did not identify themselves and their answers were only included in the sample if informed consent was provided before the survey was administered. All procedures followed national legislation and the Declaration of Helsinki. Adults (aged 18 years or older) that responded to all survey questions were included in our analyses.

### Procedures

A 70-item questionnaire was developed by senior researchers with specialties in public health, nutrition, physiology, kinesiology, neuroscience, human behavior theory. The instrument was divided into 7 domains: (a) personal information; (b) COVID-19 personal care; (c) physical activity; (d) eating behavior; (e) health risk habits; (f) mental health; (g) overall health. For the purposes of the present study, only the following domains were analyzed: (a) personal information, (b) COVID-19 personal care, (c) physical activity, (e) health risk habits, (f) mental health, and (g) overall health domains were analyzed (Diniz et al., 2020).

### Eating Behavior

In order to explore the possible impacts of COVID-19 on respondents' eating habits, the following question was asked: “Due to the COVID-19 pandemic, did your fruit intake increase?” This same question was also asked in relation to the consumption of vegetables, fried foods and sweets. Answers were categorized, such that those who answered “Nothing” or “Little” were classified as “COVID-19 did not impact eating habits.” Responses recorded as “Moderate” or “Highly” were classified as “COVID-19 impacted eating habits.” Increased intake of fruits and vegetables during quarantine were classified as, “Increased healthy food intake,” while increases in the intake of fried or sweet foods were classified as, “Increased consumption of unhealthy foods.”

A cluster of eating habits was then created, and the difference between the sum of healthy eating habits less the sum of unhealthy eating habits, was calculated: Example: [(increased consumption of fruit + increased consumption of vegetables)—(increased consumption of sweets + increased consumption of fried foods)]. When the calculation resulted in a positive value, this indicated there was an overlap of healthy eating habits over unhealthy ones, however in the negative case the opposite occurred. For example, the participant who increased the consumption of fruits and vegetables (two habits considered healthy) and increased only one of the habits considered unhealthy (increased consumption of sweets or fried foods) would have a score of 1 (2 healthy habits −1 habit not healthy = score + 1).

### Physical Activity

In order to assess physical activity habits, participants were asked: (a) “How many times are you exercising per week?” (possible answers ranged from: “none” to “7 days a week”); (b) “For how long are you exercising?” (possible answers: “none”; “ <30 min”; “between 30 and 60 min,” and; “more than 60 min”); (c) “For how long have you engaged in this physical activity?” (possible answer: “ <1 month”; “between 1 and 3 months”; “between 3 and 6 months”; “more than 6 months,” and; “I am not exercising”); (d) “What is the intensity of the physical activity?” (possible answers: “low—i.e., to bathe, to shave, to drive, wash the dishes, make the bed”; “medium/moderate—i.e., gardening, play volleyball, water aerobics, pedal, brisk walking”; “high—i.e., climb stairs, swimming, jump rope, play soccer, running,” and; “I am not exercising”); (e) “What type of exercise are you doing?” (possible answers: “walking/jogging”; “resistance training”; “core exercise”; “I am not exercising”; “others”—open response option).

Based on these responses, time spent during each exercise session during the week was multiplied by the number of days spent exercising each week. For the transformation of physical activity into a continuous variable, we consider at least the minimum of physical activity that the participant reported that he did on the days of the week. For example, if a participant answered that he or she performed physical activity for 30–60 min, we determined that at least 30 min of activity was performed. We then multiplied 30 min by the frequency of physical activity reported for the week.

Those that reached 150 min or more of moderate-vigorous physical activity (MVPA) were considered, “physically active” whereas those that fell below this threshold were classified as “inactive”^1^.

Therefore, physical activity was categorized accordingly: (a) 0–30 min/MVPA; (b) 31–90 min/MVPA; (c) 91–150 min/MVPA; (d) 151–300 min/MVPA; and (e) >300 min/MVPA.

### Covariates: Personal Information, Anthropometry, and Social Isolation Status

Some self-reported data were also collected and included as covariates when performing the One-Way ANOVA analyses: (a) Sex (“Woman or Man”); (b) Date of birth (DD/MM/YYYY); (c) Education level: “What is your level of education?” (possible responses: “elementary school,” “high school,” “college,” and “postgraduate”); (d) Anthropometric variables were assessed by self-report of weight (kg) and height (m). Body mass index (BMI) was calculated by dividing body weight by height squared; (e) Participants were also asked, “How long have you experienced social isolation?” with an open response option.

### Statistical Analysis

Data were exported from Google Forms into MS Excel v. 2019 (Redmond, WA, USA), and ultimately imported into the Statistical Package for the Social Sciences (SPSS) version 26 (IBM, Armonk, NY, USA) for analysis.

Means and standard deviations of the study sample's characteristics were calculated (see Table 1). Associations between physical activity and eating habits were analyzed using the chi-square test. The magnitude of the associations between physical activity and eating habits were assessed via binary logistic regression using the raw and adjusted model (sex, age, education level, and social isolation). Subsequently, clusters using the sum of “increased consumption of healthy foods” (fruits + vegetables) and “unhealthy foods” (sweets + fried foods) were created. The score variation ranged from zero to two. After the creation of these two clusters, the difference in the “healthy eating habits” cluster was calculated by subtracting from it the “unhealthy eating habits” cluster. A comparison of differences in the eating habits clusters and physical activity levels [(a) 0–30 min/MVPA; (b) 31–90 min/MVPA; (c) 91–150 min/MVPA; (d) 151–300 min/MVPA and (e) >300 min/MVPA] was performed using a One-Way Analysis of Variance (ANOVA). An approximate value when performing this calculation was used. For example, if a participant answered that he or she performed physical activity for 30–60 min, we determined that at least 30 min of activity was performed. We then multiplied 30 min by the frequency of physical activity reported for the week.

**Table 1:** Comparison of the characteristics of physical inactive and active Brazilians who answered an online survey during the COVID-19 social isolation, *n* = 1,874.

**Variables**	**Inactive *n =* 1,339**	**Active *n =* 535**	***p***
Age (years)	38.19 (12.81)	38.59 (13.77)	0.555
Weight (kg)	73.71 (16.52)	73.18 (16.57)	0.537
Height (cm)	167.21 (13.38)	168.52 (13.71)	0.059
Body mass index (kg/m^2^)	25.83 (4.54)	25.17 (4.31)	0.008
**Sex (%)**			
Men	39.2	46.7	0.003
Women	60.8	53.3	
Social isolation (% more than 15 days)	91.8	92.1	0.875
**Education (%)**			
Elementary school	0.3	0.4	
High school	8.5	7.7	0.441
College	41.4	45.4	
Postgraduate	49.8	46.5	

*Data showed as mean (standard deviation) or relative frequency (%)*.

Bonferroni's *post-hoc* test was used to detect possible differences. Statistical significance was set at 5% with a confidence interval of 95%.

## Results

Of those who completed the online questionnaire, 1,874 (1,099 of which identified as women) met the inclusion criteria. A total of 1,339 were classified as physically inactive (71.5%) compared to 535 that were determined to be physically active (≥150 min in MVPA per week).

Table 1 compares the characteristics of those determined physically inactive and physically active. The physically active group consisted of a higher percentage of women (53.3%, *p* = 0.003) and lower BMI (*p* = 0.008). There were no differences between age, weight, height, and social isolation (in days).

Increased fruit consumption during quarantine was observed in 25.9% of the participants, being marginally higher in those identified as being physically active (≥150 min in MVPA per week) (*p* = 0.076). Increased vegetable consumption was reported by 26.6% of the participants. Regarding unhealthy habits, 18.8% of all respondents reported increasing their consumption of fried foods during COVID-19. However, those labeled inactive reported this more frequently (*p* < 0.001). Increased consumption of sweets was reported most often reported change, with 42.5% of respondents reporting this behavior. Those identified as physically inactive reported this behavior more frequently when compared to those in the physically active group (*p* < 0.001).

Table 2 shows the associations between physical activity practice during COVID-19 and the consumption of fruits, vegetables, fried foods and sweets. During the quarantine, physically active participants were 40 and 47% less likely to increase consumption of fried foods and sweets during COVID-19, respectively.

**Table 2:** Association between physical activity (≥ 150 min in MVPA) and change in food habits during COVID-19 social isolation in Brazilians, *n* = 1,874.

	**Moderate/Vigorous physical activity**					
		**Crude**			**Adjusted**	
	**Odds ratio**	**95%CI**	***p-*value**	**Odds ratio**	**95%CI**	***p-*value**
Increase fruits consumption	1.18	0.94–1.48	0.143	1.24	0.98–1.56	0.076
Increase vegetables consumption	1.16	0.93–1.44	0.188	1.25	0.99–1.58	0.055
Increase fried food consumption	**0.58**	**0.44–0.78**	** <0.001**	**0.60**	**0.44–0.81**	** <0.001**
Increase sweet consumption	**0.49**	**0.40–0.61**	** <0.001**	**0.53**	**0.42–0.66**	** <0.001**

*Adjusted by sex, age, education level, social isolation, and BMI. Results with p < 0.05 are presented in bold*.

**Figure 1:**
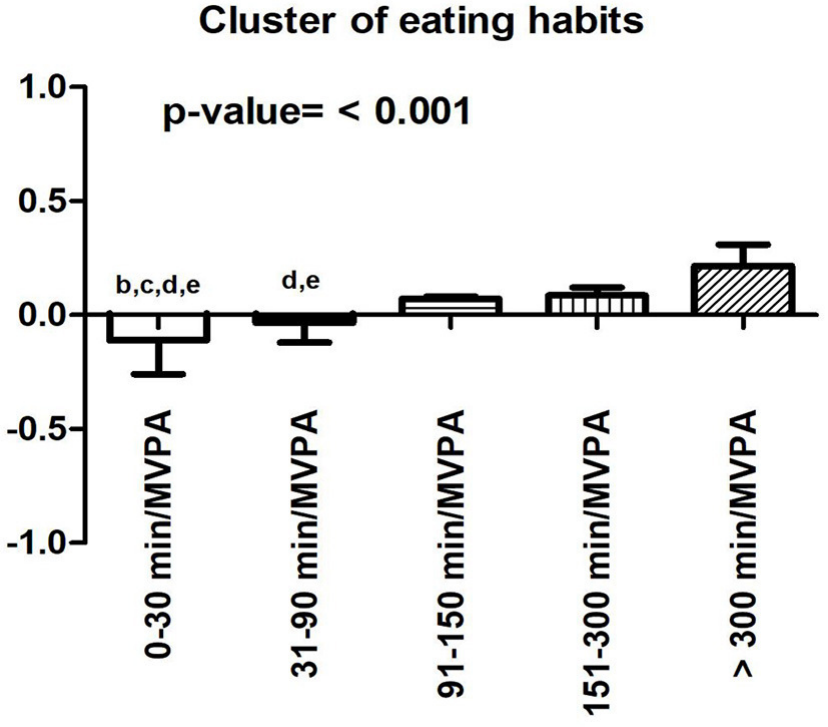
Comparison of the food cluster during quarantine in COVID-19 in inactive and active participants. b, difference with 31–90 min/MVPA; c, difference with 91–150 min/MVPA; d, difference with 151–300 min/MVPA; e, difference with >300 min/MVPA. Adjusted by sex, age, education level, social isolation and BMI.

The number of participants in each of the BP categories was as follows: 0–30 min (*n* = 710; 37.9%); 31–90 min (*n* = 433; 23.1%), 91–149 min (*n* = 195; 10.4%); 150–300 min (*n* = 476; 25.4%); <300 min (*n* = 60; 3.2%). More time spent participating MVPA was associated with better eating habits. This information is shown in Figure 1.

## Discussion

In summary, the main findings of this study were: (i) ~30% of the participants were engaged in some form of physical activity; (ii) during the data collection period, women reported being more physically active than men; (iii) increased consumption of sweets was the most frequently reported change in eating habits during the COVID-19 pandemic; and (iv) physical activity was associated with reduced intake of fried foods and sweets.

Participation in regular physical activity is important, particularly during a the current COVID-19 pandemic. Preliminary research has shown that regular physical activity during COVID-19 has been essential for maintaining health (Chen et al., 2020a). We discovered the prevalence of moderate to vigorous physical activity was higher in women. The vast majority of the literature has reported that men are more likely to engage in regular physical activity (Fernandes et al., 2010; Christofaro et al., 2019). One of the reasons for these findings may be due to history effects. In May 2020, in response to social distancing requirements, gyms were closed and activities such as team sports and football, which are widely practiced in Brazil were restricted. Men most often participate in these forms of physical activity. In addition, one of the study hypotheses is that moderate intensity physical activity related to domestic services (American College of Sports Medicine., 2018) were increased, which disproportionately affect women and lead to increased physical activity levels within this group, specifically. In addition, previous studies show that the practice of physical activity has been related to better eating habits (Pavičić ŽeŽelj et al., 2019), which seems to have been confirmed during quarantine, as indicated by the results of the present study. This relationship could perhaps be caused by the fact that women are more concerned with health when compared to men and because they often concerned about health status (Werneck et al., 2020).

Of note, periods of confinement and the associated reduction in physical activity levels compounded by increased daily calorie intakes may contribute to an increased risk of metabolic disease (Martinez-Ferran et al., 2020). In the present study, we showed that participants may increase their intakes of fried and sweet foods which are not ideal for promoting optimal health during the current pandemic. Increased consumption of fried and sweet foods may increase metabolic risks, such as the development of obesity and Type 2 diabetes (Gross et al., 2014; Qi et al., 2014; Crovetto et al., 2018). One reason for this type of behavior is that a COVID-19 pandemic can cause stress, mainly due to confinement and social isolation. Torres and Nowson (2007) showed that stress can alter food intake and subsequently contribute to obesity. Mattioli et al. (2020) also discovered that anxiety during the quarantine could contribute to the purchase of large quantities of processed foods (for fear of a possible food shortage), which generally has a longer shelf-life, but is higher in salt and sugar, which could contribute to increasing obesity levels. Quarantining can also contribute to other obesogenic behaviors, such as increased desire to consume sweets and alcohol (Tebar et al., 2021).

Such findings are concerning, as several studies have shown poor COVID-19 prognoses in patients with obesity. Chen et al. (2020b) and Simonnet et al. (2020) report obese patients experience greater severity of symptoms COVID-19. Klang et al. (2020) in a study of more than 3,000 participants showed that hospitalized patients under the age of 50 with morbid obesity were more likely to die from COVID-19.

Another interesting finding of the present study is that, as MVPA time increased during the week, there was an improvement in the cluster of eating habits, indicating a linear relationship between physical activity and quality of food intake during the pandemic. Similar to our findings, Pavičić ŽeŽelj et al. (2019) studied 400 adults in Croatia and observed that those who performed more than 150 min of moderate-vigorous physical activity had healthier eating habits (specifically, greater adherence to the Mediterranean diet which emphasizes the consumption of minimally-processed and nutrient-dense foods such as olive oil, fruits, vegetables, whole grains, lean meats, beans, lentils, milk, and cheese). It should be noted that the associations between physical activity and eating habits we observed may be partially explained by other factors. The confinement period can trigger episodes of anxiety (Bhutani and Cooper, 2020; Pappa et al., 2020), which could contribute to the consumption of high-calorie foods (Sominsky and Spencer, 2014). However, physical activity may reduce feelings of anxiety during quarantine and subsequently decrease the chances of consuming unhealthy foods (Such et al., 2020).

Global organizations suggest that maintaining physical activity levels during the pandemic is necessary^1^. However, considering the risks of participating in physical activity outside the home due to COVID-19, simple strategies such as encouraging walking near the home (while incorporating appropriate prevention measures) or virtual classes within the home conducted by Physical Education professionals can be considered good options during this critical period. In this sense, practicing regular physical activity even during the COVID-19 pandemic can positively influence other lifestyle habits (Natalucci et al., 2020), including eating habits. In turn, this may reduce the spread and/or development of complications due to COVID-19.

The present study has some limitations. The cross-sectional design prevents any determination of cause-and-effect between participation in regular physical activity and eating habits. Additionally, more women than men completed the survey, which reduces our ability to generalize these results. Another potential limitation is that the questionnaire did not provide an option for respondents to report decreased food intakes Finally, all measures were self-reported and the physical activity measurement tool was not previously validated. However, the large sample size and the fact that these data examined health behaviors during social isolation and widespread quarantine, may help us understand how behaviors may change during such an unprecedented period in time.

The findings of the present study highlight the importance of engaging in regular physical activity during the recent pandemic, since physical inactivity may contribute to the ingestion of sweets and fried foods. While engaging in regular physical activity during the pandemic may not seem feasible initially, it can be possible with even at home and respecting the protocols of social distancing. Health promotion actions aiming at increasing/maintaining physical activity and the consumption of fresh foods and reduction of ultra-processed foods must be developed and tested, especially in times of pandemic in which these problems may be accentuated.

## Conclusion

Based on the findings of the present study, the practice of physical activity was positively related to healthier eating habits during COVID-19. The importance of physical activity must be emphasized for the maintenance of health during a global pandemic while adhering to public health guidelines for preventing transmission of the virus. It may be worthwhile for policymakers and health professionals to continue promoting regular physical activity among the general public during the pandemic.

## Data Availability Statement

The raw data supporting the conclusions of this article will be made available by the authors, without undue reservation.

## Ethics Statement

This study was approved by the Universidade Nove de Julho' Ethics Committee prior to data collection (CAAE #30890220.4.0000.5511). Participants did not identify themselves and their answers were only included in the sample if informed consent was provided before the survey was administered. All procedures followed national legislation and the Declaration of Helsinki. Adults (aged 18 years or older) that responded to all survey questions were included in our analyses. The patients/participants provided their written informed consent to participate in this study.

## Author Contributions

MC, RR-D, and WP: project design. DC, AW, and WT: analysis and data interpretation. DC, AW, RR-D, and WP: article writing. ML-P, MC, JB, GC, and NM: final approval of the version to be published and important intellectual contributions. GC: conception and design. All authors contributed to the article and approved the submitted version.

## Conflict of Interest

The authors declare that the research was conducted in the absence of any commercial or financial relationships that could be construed as a potential conflict of interest.

## Footnotes

^1^Available online at: http://www.who.int/news-room/campaigns/connecting-the-world-to-combat-coronavirus/healthyathome/healthyathome---physical-activity.
